# Arterial Pulsations cannot Drive Intramural Periarterial Drainage: Significance for *A*β Drainage

**DOI:** 10.3389/fnins.2017.00475

**Published:** 2017-08-24

**Authors:** Alexandra K. Diem, Matthew MacGregor Sharp, Maureen Gatherer, Neil W. Bressloff, Roxana O. Carare, Giles Richardson

**Affiliations:** ^1^Computational Engineering and Design, Faculty of Engineering & the Environment, University of Southampton Southampton, United Kingdom; ^2^Clinical Neurosciences, Faculty of Medicine, University of Southampton, Southampton General Hospital Southampton, United Kingdom; ^3^Mathematical Sciences, Faculty of Social, Human and Mathematical Sciences, University of Southampton Southampton, United Kingdom

**Keywords:** Alzheimer's disease, cerebral blood flow, perivascular drainage, intramural periarterial drainage, cerebral lymphatics

## Abstract

Alzheimer's Disease (AD) is the most common form of dementia and to date there is no cure or efficient prophylaxis. The cognitive decline correlates with the accumulation of amyloid-β (*A*β) in the walls of capillaries and arteries. Our group has demonstrated that interstitial fluid and *A*β are eliminated from the brain along the basement membranes of capillaries and arteries, the intramural periarterial drainage (IPAD) pathway. With advancing age and arteriosclerosis, the stiffness of arterial walls, this pathway fails in its function and *A*β accumulates in the walls of arteries. In this study we tested the hypothesis that arterial pulsations drive IPAD and that a valve mechanism ensures the net drainage in a direction opposite to that of the blood flow. This hypothesis was tested using a mathematical model of the drainage mechanism. We demonstrate firstly that arterial pulsations are not strong enough to produce drainage velocities comparable to experimental observations. Secondly, we demonstrate that a valve mechanism such as directional permeability of the IPAD pathway is necessary to achieve a net reverse flow. The mathematical simulation results are confirmed by assessing the pattern of IPAD in mice using pulse modulators, showing no significant alteration of IPAD. Our results indicate that forces other than the cardiac pulsations are responsible for efficient IPAD.

## 1. Introduction

Alzheimer's disease (AD) is the commonest form of dementia. Although it has been studied for over 100 years (Selkoe, 2001), to date there is no cure as the processes relevant to its onset and progression have not yet been fully understood. The pathology of AD comprises the degeneration and death of neurons, synapse loss, neuroinflammation and the intracellular as well as extracellular accumulation of proteins (Koffie et al., 2011). The protein amyloid-β (*A*β) is produced as part of the normal metabolism of healthy brains, but it plays a major role in the development of AD (Haass et al., 1992). According to the *A*β hypothesis AD is caused by a failure to remove *A*β from the brain and successively, its accumulation in the form of plaques in the parenchyma. Prior to the buildup of plaques in the brain *A*β accumulates in the walls of cerebral blood vessels as cerebral amyloid angiopathy (CAA), which correlates much better with the degree of dementia compared to the number and size of plaques and appears in 90–96% of AD patients (Weller et al., 2006, 2009).

In order to process metabolites, the brain requires a lymphatic system that transports waste products to the lymph nodes. While the rest of the body possesses a dedicated network of lymphatic vessels these do not exist in the brain (Weller et al., 2010). Both the pathology of CAA and ample evidence from injection studies suggest that the basement membranes (BM) in the walls of cerebral arteries provide the clearance pathway for interstitial fluid (ISF) toward the surface of the brain, also referred to as intramural periarterial drainage (IPAD) (Carare et al., 2008; Weller et al., 2010; Hawkes et al., 2011; Morris et al., 2014). It is important not to confuse the IPAD pathway with the Virchow-Robin spaces, which are often referred to as perivascular (or “paravascular”) spaces, possibly artifactual, located between the glia limitans and the arterial wall. Virchow-Robin spaces are compartments occupied by the pial-glial basement membranes spaces and have no role in *A*β drainage from the extracellular spaces. The BM forming the IPAD does not actually constitute a space, but rather a fluid-filled protein matrix. Our understanding of the mechanism that drives perivascular drainage through the BM is still very limited. The evidence indicates that ISF enters the BM at the capillary level and flows toward the lymph nodes in the neck via the BM cerebral arteries. This suggests that the counterintuitive phenomenon of ISF flow occurs in the reverse direction to blood flow (Carare et al., 2008).

A widely accepted hypothesis for the driving mechanism of this reverse perivascular drainage of solutes from the brain is that it is driven by arterial pulsations (Schley et al., 2006; Carare et al., 2008; Weller et al., 2009, 2015; Attems et al., 2011; Hawkes et al., 2011; Wang and Olbricht, 2011; Iliff et al., 2012; Morris et al., 2014; Asgari et al., 2015; Sharp et al., 2015). In this paper we tested this hypothesis by using a multi-scale modeling approach combining analytical and numerical methods. The BM is modeled as a porous medium using a lubrication approximation. Flow inside the BM is driven by both the ISF pressure gradient and the deformation of the arterial wall, which are obtained from a numerical model of the middle cerebral artery (MCA). To give IPAD in the reverse direction to the blood flow, a valve mechanism is required; here it is modeled by a pressure gradient dependent permeability. It is shown that this valve mechanism is necessary to achieve net reverse drainage. However, due to the long wavelength of arterial pulsations the pressure gradients inside the BM are too small to drive fluid flow at the expected velocities. The resulting velocity of the model was at least four orders of magnitude smaller than values obtained experimentally (Carare et al., 2008). Therefore, arterial pulsations are concluded to be insufficient to drive perivascular drainage along the BM of cerebral arteries. The simulation results are confirmed by an experimental assessment of the effect of pulse modulation in a mouse model, which showed no significant difference in IPAD compare to control mice.

## 2. Numerical modeling of perivascular drainage of *A*β

First, IPAD under a pressure-pulse driven scenario is evaluated using computational modeling. An analytical model of the BM was developed, which captures the preference of flow in the reverse direction to the blood flow. Pressure inside the BM is evaluated using a numerical model of blood pressure inside the artery and a stress analysis in the artery wall. These were then used to evaluate the velocity of perivascular drainage through the MCA.

### 2.1. Porous medium model of lymphatic drainage through the BM

We propose a model of lymphatic drainage from the brain via the cerebral BM based on Darcy's law for flow in porous media. By exploiting the discrepancy of scales between the width of the BM and its length we approximate the flow by a one-dimensional lubrication model (see for example Ockendon and Ockendon, 1995). Furthermore, we allow the permeability of the BM to depend on the pressure gradient of the ISF. Such a pressure gradient dependent permeability could, for instance, model proteins in the BM protein network that have structural properties that allow them to bend easily only in one direction, thereby acting as a valve, and thus provide a higher resistance to flow in the direction of the blood flow. A similar idea has been investigated in Sharp et al. (2015), where proteins were explicitly modeled as cilia. However, here we model the active component of the BM generically in this model and thus allow for alternative explanations of the phenomenon.

The BM consists of a complex matrix of proteins (extracellular matrix, ECM) that can be interpreted as a porous medium through which soluble metabolites drain via the ISF. We model flow through this medium, in the standard fashion, by Darcy's law in a 2D cylindrical coordinate system. Darcy's law states that the flux *q* is related to the fluid pressure gradient **∇***p* by

(1) $$\begin{array}{l} q=-\frac{k}{\mu }\nabla p \end{array}$$

where *k* is the intrinsic permeability of the ECM and μ is the viscosity of ISF. Since ISF flows up the artery, counter to the direction of arterial pulsations, a valve-like mechanism in the ECM is required if arterial pulsations are indeed the motive force for this flow. This valve-like property of the ECM can be modeled by a Darcy flow Equation (1), in which the intrinsic permeability is a function of the axial pressure gradient ∂*p*/∂*z* along the vessel. On assuming an artery aligned along the *z*-axis with net blood flow and arterial pulsations propagating the positive *z*-direction we model valve-like properties of the ECM in the BM by a pressure gradient dependent permeability

(2) $$\begin{array}{l} k=\mu K\left(p_{z}\right)\text{ where }p_{z}=\frac{\partial p}{\partial z}. \end{array}$$

In order to obtain a net flux up the vessel, counter to the blood flow, we require this function to be increasing in *p*_*z*_, representing the notion that it is harder to push a flow in the positive *z*-direction (*p*_*z*_ < 0 and *K*(*p*_*z*_) small) than in the negative *z*-direction (*p*_*z*_ ≥ 0 and *K*(*p*_*z*_) large).

The governing equations in cylindrical coordinates are thus

(3) $$\begin{array}{l} q=q_{1}{\text{e}}_{\text{z}}+q_{2}{\text{e}}_{\text{r}}=-K\left(p_{z}\right)\left({\text{e}}_{\text{z}}p_{z}+{\text{e}}_{\text{r}}p_{r}\right) \end{array}$$

(4) $$\begin{array}{l} \frac{\partial q_{1}}{\partial z}+\frac{1}{r}\frac{\partial }{\partial r}\left(rq_{2}\right)=0, \end{array}$$

where **e**_**z**_ and **e**_**r**_ describe the unit vectors in the *z* and *r* direction. Incompressibility of the fluid is represented by Equation (4).

**Figure 1:**
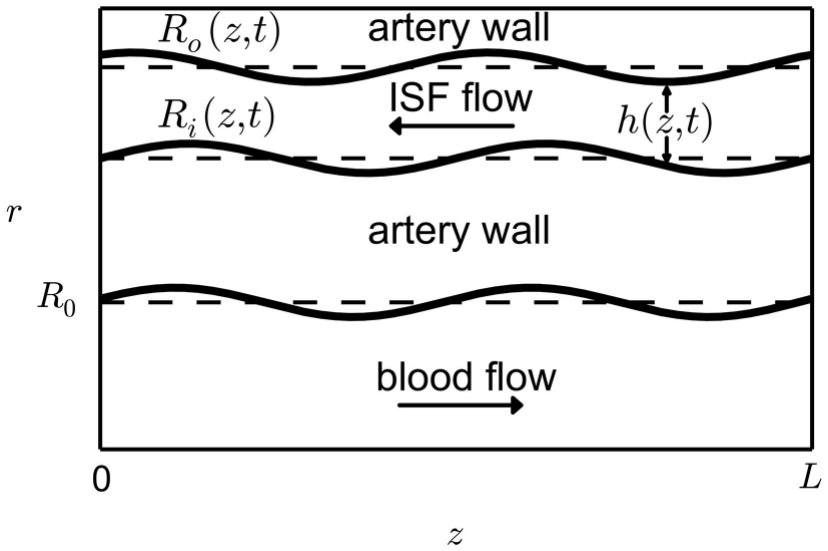
Geometry of the perivascular drainage pathways model. The length *L* of a typical artery in the brain can be assumed to be much greater than the width *W* of the perivascular drainage pathways. Therefore we can approximate the flow as a lubrication model. The pulsation of the capillary is described by the function *h*(*z, t*).

To determine the boundary conditions of the system consider the geometry in Figure 1. Under the assumption that the vessel deformations are radially symmetric the positions of the inner and outer sheaths bounding the BM can be described by the two expressions *r* = *R*_*i*_(*z, t*) and *r* = *R*_*o*_(*z, t*), respectively. Assuming that the flows through the surfaces of the BM are negligible the following kinematic boundary condition applies

(5) $$\begin{array}{l} \frac{D}{Dt}\left(r-R_{i}\left(z,t\right)\right)=0\text{ on }r=R_{i}\left(z,t\right) \end{array}$$

and analogously for *R*_*o*_(*z, t*).

Because the BM thickness is much smaller than the vessel length it is possible, on using the lubrication approximation, to derive a 1D model (in *z*) for the BM thickness *h*_bm_(*z, t*); this takes the form

(6) $$\begin{array}{l} \frac{\partial }{\partial t}\left(\gamma R_{i}\left(z,t\right)\cdot h_{\text{bm}}\left(z,t\right)\right)=\frac{\partial }{\partial z}\left(R_{i}\left(z,t\right)\cdot h_{\text{bm}}\left(z,t\right)\cdot K\left(p_{z}\right)\cdot p_{z}\right), \end{array}$$

where γ is the fluid volume fraction in the BM (see also Supplementary Material 1). Equation (6) describes the evolution of the BM thickness and can be solved using a finite difference approximation. For a detailed derivation see Supplementary Material 1. Another method to arrive at the same equation is derived by considering an infinitesimally small volume of BM *dV* = 2π · *R*_*i*_(*z, t*) · *h*_bm_(*z, t*) · *dz*. Then the difference between the flux into and out of the element *dV* is equal to the rate of change of the fluid volume ∂(γ*dV*)/∂*t*, which can be written as

(7) $$\begin{array}{l} \frac{\partial }{\partial t}\left(\gamma R_{i}\left(z,t\right)\cdot h_{\text{bm}}\left(z,t\right)\cdot dz\right)=R_{i}\left(z,t\right)\cdot h_{\text{bm}}\left(z,t\right)\cdot q_{1}\left(z,t\right)\\ -\;R_{i}\left(z+dz,t\right)\cdot h_{\text{bm}}\left(z+dz,t\right)\\ \cdot \;q_{1}\left(z+dz,t\right), \end{array}$$

which is equal to the model Equation (6) in the limit *dz* → 0. The pressure *p*(*z, t*) and the displacement of the BM wall *R*_*i*_(*z, t*) are, to a very good approximation, determined solely by arterial pressure (i.e., almost independent of the BM flow), see Supplementary Material 2 for more details. *R*_*i*_(*z, t*) and *p*_*z*_ are obtained from a numerical artery model described in the next section. To model the protein valve that is being proposed here, *K*(*p*_*z*_) is defined as a step function

(8) $$\begin{array}{l} K\left(p_{z}\right)=\left\{ \begin{array}{cc} K_{0}\; & p_{z}<0\\ K_{1}\; & p_{z}\geq 0, \end{array}\right. \end{array}$$

such that *K*_1_ > *K*_0_ > 0 so that the BM is more permeable to ISF flow in the negative *z*-direction than in the positive one.

### 2.2. Numerical model of a straight arterial section

In order to obtain the input functions *R*_*i*_(*z, t*) and *p*_*z*_ for the analytical model developed in the previous section, a section of human MCA was modeled as a 1D axisymmetric tube in a cylindrical coordinate system with cross-sectional lumen area *A*(*z, t*) and flux *Q*(*z, t*) using the Python package VaMpy (Diem and Bressloff, 2016). The geometry parameters are listed in Table 1. Blood flow through arteries and the propagation of arterial pulsations are commonly described by a one-dimensional averaged model (Olufsen et al., 2000) (for more details see Diem, 2016). It is represented by the conservation law

(9) $$\begin{array}{l} \frac{\partial U}{\partial t}+\frac{\partial F}{\partial z}=S, \end{array}$$

where

$$\begin{array}{l} U=\left(\begin{array}{c} A(z,t)\\ Q(z,t) \end{array}\right),\;F=\left(\begin{array}{c} Q(z,t)\\ \frac{Q{\left(z,t\right)}^{2}}{A(z,t)}+f(r_{0})\sqrt{A_{0}(z)A(z,t)} \end{array}\right),\;S=\left(\begin{array}{c} 0\\ S_{1} \end{array}\right)\\ S_{1}=-\frac{2\pi R(z,t)}{\delta_{b}\text{Re}}\frac{Q(z,t)}{A(z,t)}+(2\sqrt{A(z,t)}\left(\sqrt{\pi }f(r_{0})+\sqrt{A_{0}(z)}\frac{df(r_{0})}{dr_{0}}\right)\\ \;-A(z,t)\frac{df(r_{0})}{dr_{0}})\frac{dr_{0}(z)}{dz}. \end{array}$$

**Table 1:** Geometry parameters of the MCA section modeled using VaMpy (Diem and Bressloff, 2016).

	**MCA (cm)**	**Left daughter vessel (cm)**	**Right daughter vessel (cm)**	
*R*_*u*_	0.14	0.13	0.13	Upstream radius
*R*_*d*_	0.14	0.08	0.08	Downstream radius
*L*	7	6.5	6.4	Length

The equation is solved for cross-sectional area *A*(*z, t*) of the vessel lumen and flux through the vessel *Q*(*z, t*), where *R*(*z, t*) = (*A*(*z, t*)/π)^1/2^ is the lumen radius, δ_*b*_ is the boundary layer thickness and Re is the Reynolds number. Elasticity of the artery wall is modeled via the quantity *f*(*r*_0_) = 4*Eh*/(3*r*_0_), where *E* is the Young's modulus and *h* is the wall thickness and *r*_0_(*z*) is the radius at rest. The relationship *Eh*/*r*_0_ is based on compliance estimates

(10) $$\begin{array}{l} \frac{Eh}{r_{0}}=k_{1}\exp\left(k_{2}r_{0}\left(z\right)\right)+k_{3} \end{array}$$

with *k*_1_, *k*_2_, *k*_3_, see Table 2 (Olufsen et al., 2000). Blood pressure *p*(*z, t*) is related to *A*(*z, t*) and *f*(*r*_0_) via the state equation

(11) $$\begin{array}{l} p\left(z,t\right)-p_{0}=f\left(r_{0}\right)\left(1-\frac{A_{0}\left(z\right)}{A\left(z,t\right)}\right), \end{array}$$

where *p*_0_ is the diastolic pressure and *A*_0_(*z*) is the lumen radius at rest. Blood pressure provides the basis for determining ISF pressure inside the BM via the calculations in Supplementary Material 2.

**Table 2:** Simulation parameters for modeling a bifurcation of the MCA using VaMpy (Diem and Bressloff, 2016).

**Parameter**	**Value**	**Physical meaning**
*k*_1_	2.0e7 g cm^−1^ s^−2^	Wall elasticity
*k*_2_	−22.53 cm^−1^	Parameters
*k*_3_	8.65e5 g cm^−1^ s^−2^	See Equation (10) Olufsen et al. (2000) and Diem and Bressloff (2016)
ν	0.046 cm2 s^−1^	Kinematic viscosity of blood
*R*_1_	14.130 g cm^−4^ s^−1^	First resistance element
*R*_2_	7.200 g cm^−4^ s^−1^	Second resistance element
*C*_*T*_	2.4752e−3 cm^4^ s^2^ g^−1^	Compliance element
*T*	0.85 s	Cardiac cycle length
*t*_*c*_	4	Number of cardiac cycles
Δ*t*	1e−5 s	Time step size
Δ*z*	0.1 cm	Spatial step size

To derive Equation (9) it was necessary to make assumptions about the velocity profile of blood flow through an artery. Blood flow is considered pulsatile laminar and vessels are considered slightly tapered, therefore the velocity profile is assumed to be mostly flat with a thin boundary layer $\delta_{b}={\left(\nu T/\left(2\pi \right)\right)}^{0.5}$, such that δ_*b*_ ≪ *R*(*z, t*) (Olufsen et al., 2000).

This system of equations can be solved numerically using Richtmyer's two-step version of the Lax-Wendroff method, as done by others (LeVeque, 1992; Olufsen et al., 2000; Kolachalama et al., 2007). We use the Python module from Diem and Bressloff (2016) to implement a model of the MCA and its first bifurcation. All simulation parameters are listed in Table 2. The MCA geometry data was taken from Cousins and Gremaud (2014), estimating lengths by 50 · *R*_*u*_. The radii of the daughter vessels were estimated using a scaling factor of 0.91 (Cousins and Gremaud, 2014). The length of the right daughter vessel was shortened slightly to avoid reflective waves canceling one another out (Kolachalama et al., 2007). A three-element Windkessel (3WK) model is used at the outlet whose parameters were obtained from Olufsen et al. (2002).

**Figure 2:**
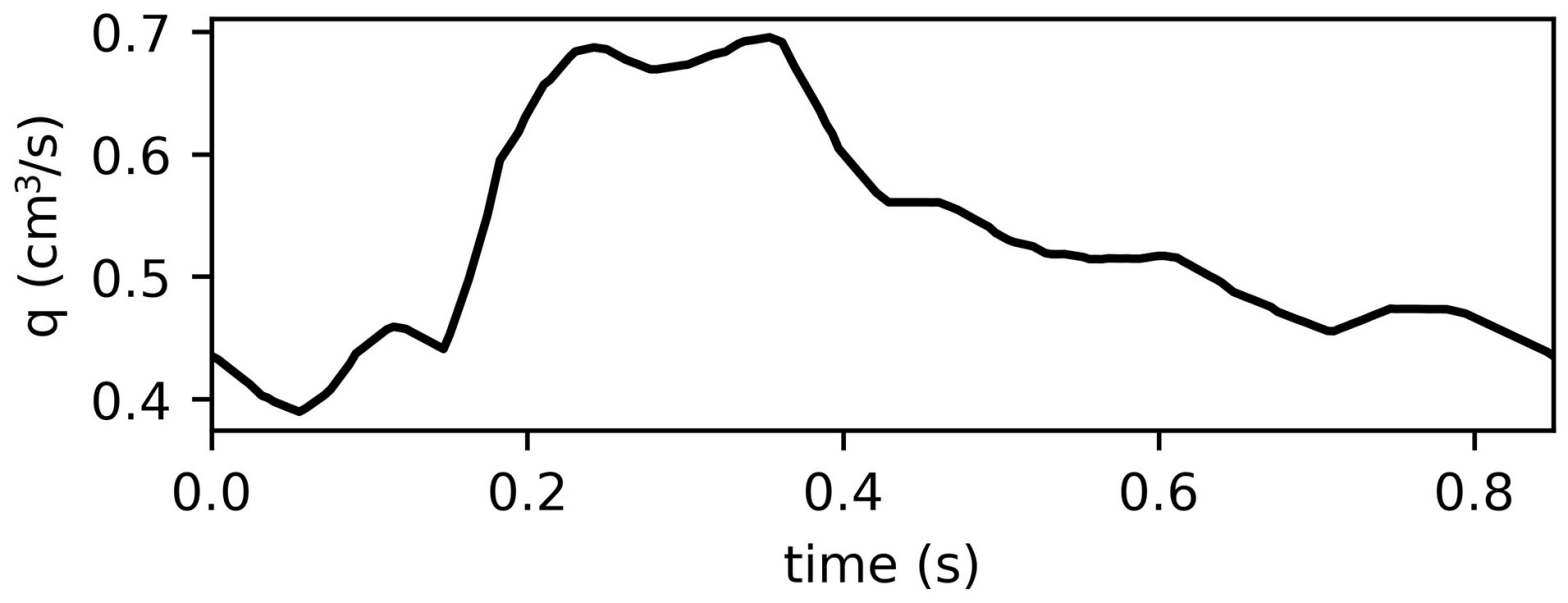
Inlet function for MCA simulations obtained from patient-specific measurements used to represent one period of 0.85 s of the pulse wave. The final 10 peaks of the velocity time series were averaged in the Fourier space and adjusted such that its range of values aligns with those reported in Olufsen et al. (2002). The resulting inlet function is smooth and serves as the inlet boundary condition for the MCA simulations using VaMpy (Diem and Bressloff, 2016).

At the inlet flux is prescribed using patient-specific flow velocity measurements collected from the MCA of a healthy adult male using Doppler Sonography ultrasound. The velocity data was converted to volumetric flux values within the range reported in Olufsen et al. (2002). To obtain a smooth inlet function the final ten peaks of the time series were averaged in the Fourier space. The resulting inlet boundary condition is shown in Figure 2.

### 2.3. IPAD through the MCA

**Figure 3:**
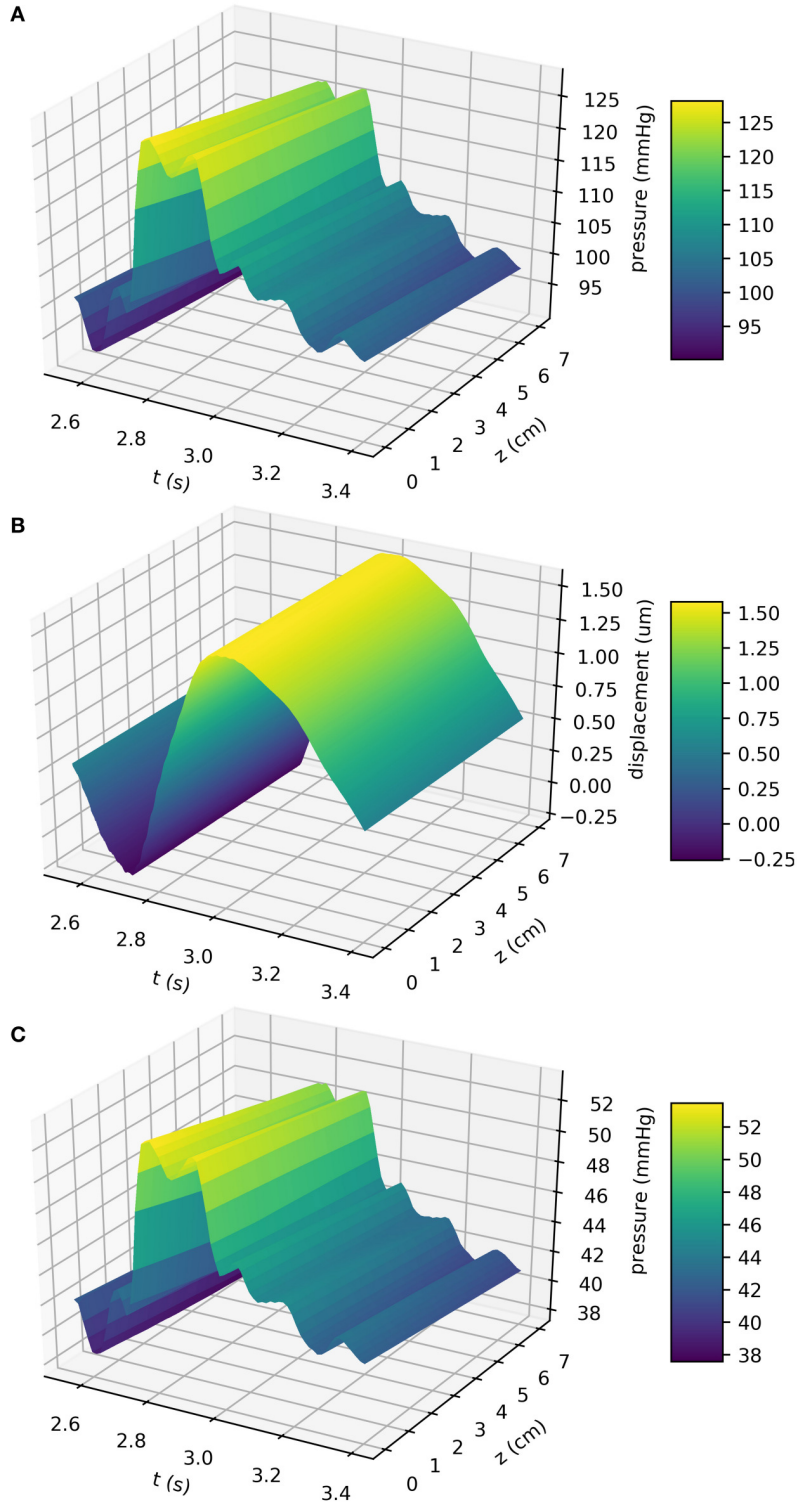
Blood pressure **(A)**, wall displacement **(B)**, and the resulting ISF pressure **(C)** in the MCA. Simulations were performed using the VaMpy Python package (Diem and Bressloff, 2016) and the simulation parameters are listed in Table 2. Wall stiffness is high in small blood vessels, therefore pressure gradients in time are steep. Wall displacement was calculated from the linear elasticity approximation derived in Supplementary Material 2. The input function *R*_*i*_(*z, t*) to the BM model Equation (6) is obtained by adding the radius at rest *a* (see geometry depiction in Figure 1).

In the previous sections the models governing blood flow through the MCA and ISF flow through the artery wall have been introduced. Here, the results of the previous sections are coupled and used to calculate ISF flow through the BM with and without a valve mechanism to show its necessity to achieve net reverse drainage. At the same time it is shown that blood pressure driven perivascular drainage is far too slow to provide any meaningful flow, thereby disproving the popular hypothesis that arterial pulsations provide the major driving force for perivascular drainage. MCA simulations were run for eight cardiac cycles to allow the system to settle. An average human heartbeat lasts 0.85 s and the total simulation time was 6.8 s (see Table 2). In the results presented here only the final heartbeat is shown. Figure 3 shows blood pressure (a), wall displacement (b), and ISF pressure (c) in the MCA. Because the radius of the MCA is very small its wall stiffness is high. Pressure gradients in time are therefore steep. This data provides the basis for estimating perivascular drainage through the cerebral vasculature.

**Figure 4:**
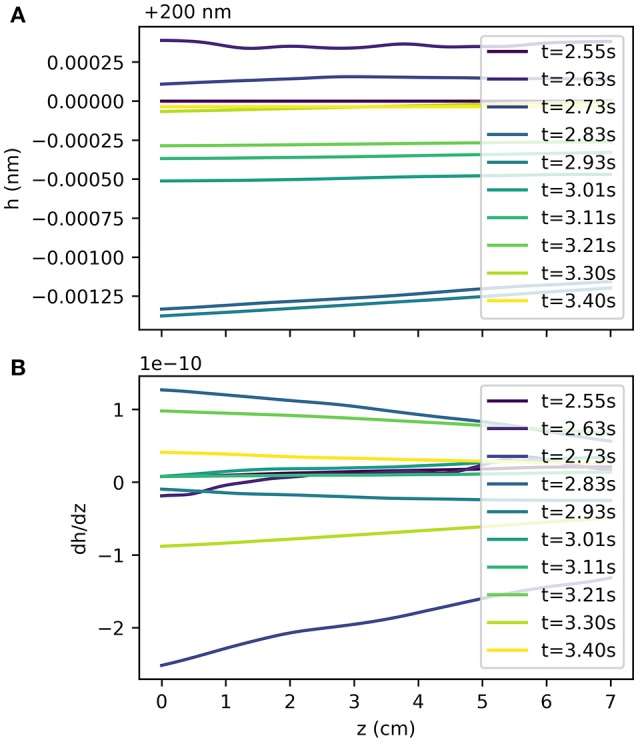
BM width *h*_bm_(*z, t*) at several time points during the cardiac cycle **(A)** and its gradient along the *z*-axis at the same time points **(B)**. Pressure pulse driven variations of the BM width are small compared to the initial width of 200 m and thus flow through the BM is slow.

Figure 3B shows the displacement of the artery wall due to the pressure pulse. To calculate the width of the BM using the model Equation (6), the input function *R*_*i*_(*z, t*) is the sum of the radius at rest and the wall displacement, as depicted in Figure 1. Figure 4A shows the width of the BM *h*_bm_(*z, t*) as calculated from Equation (6) over the length of the artery at a number of time points, while Figure 4B shows ∂*h*_bm_(*z, t*)/∂*z* for the same time points. The BM width only varies minimally around its initial width and thus flow rates of ISF are very small.

**Figure 5:**
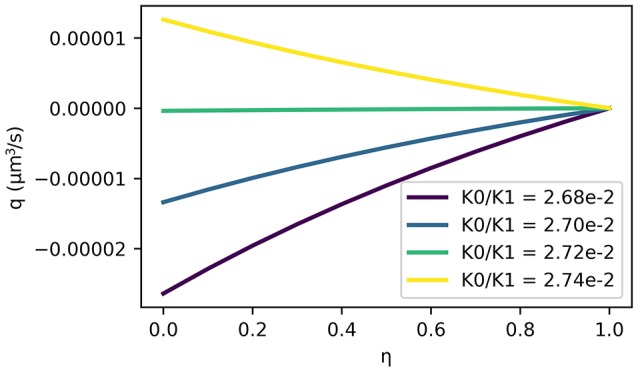
ISF flux (positive values in the direction of arterial flow) averaged over one cycle as a function of BM position η in the arterial wall. Here, the BM is at *r* = *r*_0_ + η*h* such that η = 0 represents a BM immediately adjacent to the lumen and η = 1 represents a BM on the outer wall of the artery. Note that reverse flow is only achieved by ratios of $K_{0}/K_{1}<2.74\times 10^{-2}$. The fastest net reverse drainage shown here (for $K_{0}/K_{1}=2.68\times 10^{-2}$ and on the arterial lumen η = 0) is −2.64 × 10^−5^ μm^3^ s^−1^.

The results show that a valve mechanism is required to drive perivascular drainage in the reverse direction of the blood flow with an average flux of −1.12e−3 μm3 s^−1^ for a ratio of *K*_0_/*K*_1_ = 0.01, while it is 6.33e−2 μm3 s^−1^ for *K*_0_/*K*_1_ = 1.0. While the results confirm the necessity of a strong valve mechanism under pulse driven flow they also indicate that arterial pulsations have too long a wavelength to drive significant perivascular drainage. Drainage velocity is fastest without a valve mechanism (1.81e−2 μm s^−1^, and then it is in the wrong direction), but even in that case it is almost three orders of magnitude slower than the expected value of 8.33 μm s^−1^ (Carare et al., 2008). The valve mechanism only guarantees net reverse drainage for a permeability ratio *K*_0_/*K*_1_ < 2.74e−2, where *K*_1_ is chosen such that *k* = 1e−10 cm^2^ is consistent with values for other tissues in the body and μ = 1.5e−3 Pa s (Heppell et al., 2013). Figure 5 illustrates flux through the BM for a number of ratios *K*_0_/*K*_1_ and for different positions of the BM *r* = *r*_0_ + η*h*. Reverse flow is achieved for *K*_0_/*K*_1_ < 2.74e−2. Drainage flux values are fastest when the BM is placed close to the arterial lumen (η = 0) due to the elasticity of the artery wall.

The minute flux values are a result of the very small variations in BM width (see Figure 4) over the length of the MCA, in fact they are less than 1 Å. This value is far too small to be realistic and demonstrates that arterial pulsations cannot be strong enough to drive perivascular drainage through the BM. To illustrate it in another way, the total ISF volume in a human brain is 280 ml (Weller et al., 2015). At a drainage rate of −1.12e−3 μm3 s^−1^ per cerebral artery the total turnover time for ISF would be 4.83e11 days, much more than a human life span.

## 3. Experimental assessment of intramural perivascular drainage of *A*β

In addition to performing simulations to assess the feasibility of arterial pulsations providing the main driving mechanism for IPAD, mouse model experiments were performed to confirm the results *in vivo*. Experiments on mice were carried out on 10 week old wild-type C57B16 mice (*n* = 4). All mice were kept on a standard 12 h light/dark cycle and allowed food and water *ad libitum*. All experiments were carried out in accordance with animal care guidelines stipulated by the Animal Care and Use Committee at the University of Southampton and the Home Office (PPL 20/2095).

To minimize the effects of anesthesia on mouse cardiovascular function, isoflurane was chosen as the anesthetizing agent. To reduce arterial pulsations, the beta-blocker Atenolol (Ate, 10 mg kg^−1^) was administered intraperitoneally (IP). Mouse vital parameters were monitored throughout the procedure using a MouseOX Plus and an infrared mouse thigh sensor (STARR Life Science, Holliston, MA, USA). Thermal regulation was maintained using a heat pad and rectal probe (BASi, West Lafayette, IN, USA). All intracerebral injections were performed using a 1–5μl Hirschmann microcapillary pipette (Sigma UK) with a tip adjusted to a diameter of <50 μm using a Sutter P97 Flaming Brown Pipette puller.

Mice were anesthetized with isoflurane, placed prone on a stereotaxic instrument and secured with head adaptors. Baseline measurements of oxygen saturation, heart rate, breath rate and pulse distension were taken before administering Ate. A volume of 0.5 μl of *A*β_1−40_ HiLyte Fluor 555 (Cambridge Bioscience, UK) was stereotaxically injected into the left hippocampus at a rate of 0.2 μl min^−1^ (coordinates from Bregma: AP = −1.9 mm; ML = 1.5 mm and DV = −1.7 mm). Injection pipettes were left *in situ* for 2 min to prevent reflux. Mice were euthanized 5 min after withdrawal of the injection pipette through overdose with pento-barbital (200 mg kg^−1^). Mice were then intracardially perfused with 0.01 M phosphate buffered saline (PBS), pH 7.2 followed by 4% paraformaldehyde in 0.01 M PBS, pH 7.2. Brains were removed, post fixed overnight in fresh fixative and then processed for immunohistochemistry and image analysis. For controls, 10 week old wild-type C57B16 mice were processed following the same method, but without the administration of Ate (*n* = 3).

### 3.1. Immunohistochemistry

Brains were cut into 20 μm coronal sections using a freezing microtome (Leica) and the injection site located by examining on a Zeiss Axioskop 2 fitted with a rhodamine filter. Sections 400 and 800 μm both posterior and anterior to the injection site were selected for immunohistochemistry. Sections were blocked in 15% goat serum (Sigma 9023) for 1 h at room temperature (RT). They were then incubated in primary antibodies diluted in 0.01 M phosphate buffered saline 0.1% triton x100 (PBSt). Primary antibodies comprised rabbit anti-collagen IV (AbCam, ab6586) 1/400 and mouse anti-smooth muscle actin (SMA) FITC conjugated (Sigma F3777) 1/200. Sections were incubated overnight at 4 °C in a moist chamber. Following washing 3 × 10 min 0.01 M PBS sections were incubated in goat anti-rabbit Alexa Fluor 633 (ThermoFisher Scientific A-21070) 1/200 PBSt.

### 3.2. Image analysis

Images of the whole hippocampus were captured on a Leica SP8 confocal microscope fitted with LASX software in tile scanning mode with sequential imaging. Once captured, images were exported as TIFF files and uploaded into Adobe Photoshop CS6. An area of hippocampus was drawn extending transversely from CA3 pyramidal cells to the apex of the blades of the granule cell layer and from the ventricle to *stratum oriens* in the superior/inferior axis. *A*β containing vessels were defined according to size and presence of SMA: capillaries had a diameter of ≤ 10 μm, veins had no SMA and a diameter of > 10 μm. The number of vessels was expressed per mm2 of hippocampus. Statistical analysis was performed by an independent *t*-test with a confidence interval of 95%.

### 3.3. Results

**Figure 6:**
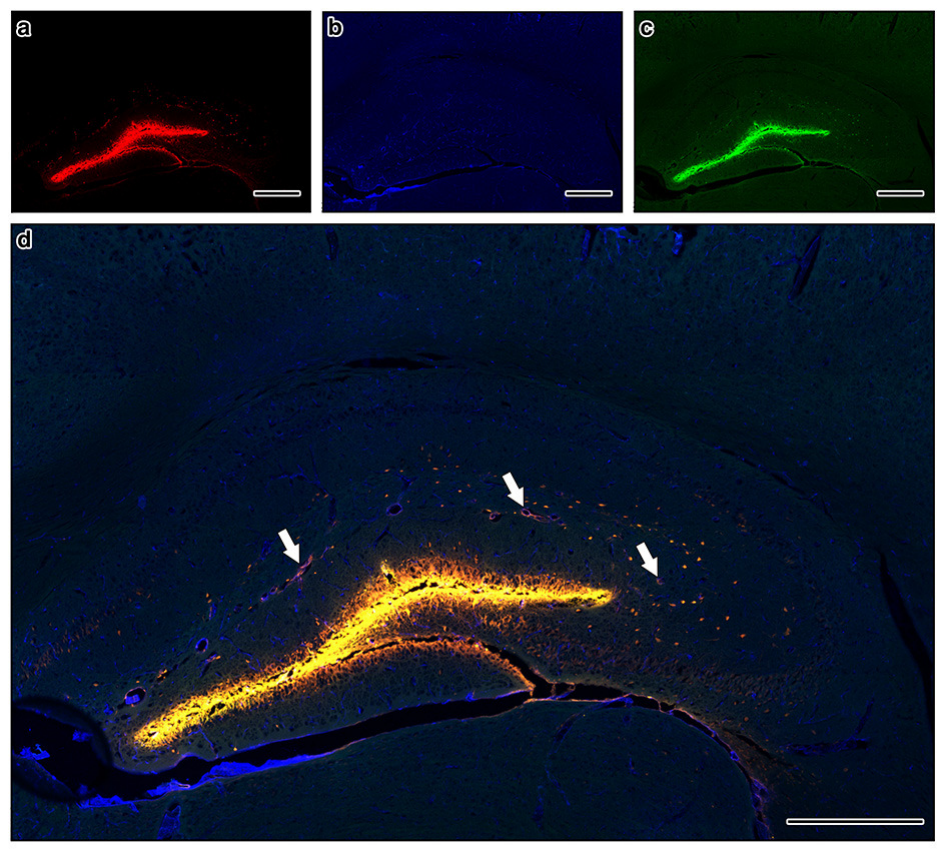
Composite tile scan of confocal images of the distribution of intracerebrally injected Aβ **(a)** in relation to collagen IV **(b)** and SMA **(c)**. The image was taken at 400 mm anterior to the injection site in an atenolol (Ate) injected mouse, to avoid any artifacts induced by the injection tract, relying instead on the pattern of distribution of the fluorescently injected *A*β away from the direct injection. Arrows on merged image **(d)** indicate arteries with *A*β in the intramural perivascular drainage pathways. Scale bars = 400 μm.

**Figure 7:**
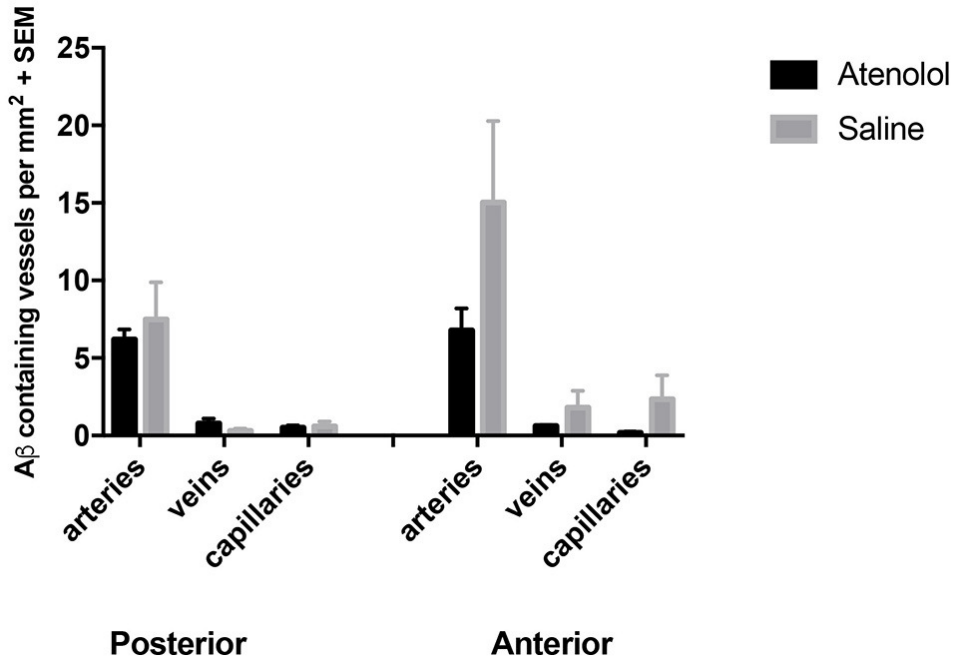
Graphs showing the number of arteries, veins and capillaries with *A*β in their walls in atenolol treated mice compared to control mice. SEM: standard error of mean.

Within 5 min of injections into the hippocampus, fluorescent *A*β was observed diffusely in the hippocampus and co-localizing with collagen IV with collagen IV in the walls of capillaries and arteries, with very few veins involved in both Ate-treated and control mice (Figure 6). Application of an independent *t*-test to the data revealed no significant differences between the saline and Ate treated mice either anterior or posterior to the injection site (Figure 7).

## 4. Discussion and conclusion

The results show that the valve mechanism as implemented in our analytical model of the BM guarantees net reverse flow inside the BM under physiological blood flow conditions. The valve mechanism is required to be strong, i.e., with a small ratio *K*_0_/*K*_1_, and necessary for net reverse flow to occur within the BM. This result agrees with previous modeling approaches that did not achieve net reverse drainage (Schley et al., 2006; Wang and Olbricht, 2011). The velocity of drainage additionally depends on the distance of the BM from the artery lumen. The closer the BM is placed to the lumen the faster net reverse drainage is, but variations remain within the same order of magnitude. This is due to the relatively large thickness of cerebral artery walls and their elastic properties. The hypothesis that the BM utilizes a unidirectional valve mechanism is plausible as the general lymphatic system of the body also has one and its existence has previously been discussed (Schley et al., 2006; Weller et al., 2010; Heppell et al., 2013).

Since the development of the Schley model we have gained knowledge on the approximate velocity of the drainage and are able to perform imaging in live mice (Schley et al., 2006; Carare et al., 2008; Arbel-Ornath et al., 2013), yet this key information has not been utilized in more recent mathematical and computational studies (Wang and Olbricht, 2011; Sharp et al., 2015). From the results of Carare et al. (2008) it was estimated that the velocity for perivascular drainage in blood vessels of roughly 10 μm in diameter is in the order of magnitude of 8 μm s^−1^. Arbel-Ornath et al. (2013) measured the dye intensity of the tracers, which does not allow them to directly measure the velocity of the drainage but, from their results, a half-life period of about 5 min can be assumed for their 3 μl injection volume. Therefore, we would expect a volumetric flux in the order of magnitude of 0.001 mm3 s^−1^. No correlation between ISF velocity and artery diameter was found.

The results from this study showed that the average drainage flux of ISF is −1.12e−2 μm3 s^−1^ at *r* = *r*_0_ + 0*h* and *K*_0_/*K*_1_ = 0.01. This value indicates a net reverse flow within the BM, although it is eight orders of magnitude smaller than the flux of 0.001 mm3 s^−1^ estimated by Arbel-Ornath et al. (2013). The results therefore suggest that arterial pulsations are not powerful enough to drive perivascular drainage of ISF through the BM. This is due to the very long wavelength of the arterial pulsations compared to the artery section considered, which results in very small pressure gradients along the vessel length (Figure 3B). These results are confirmed here by analysing the pattern of IPAD in mice after administration of pulse modulators. No significant differences were found between mice that received the pulse modulator and control mice. We were unable to measure whether the treatment of mice did not, in addition to the desired reduction in pulsation frequency, lead to a decrease in the magnitude of pulsations. However, given that the flow rate calculated from the computational model is eight orders of magnitude smaller than what would have been expected it is highly unlikely that the administration of the beta-blocker caused a pressure drop large enough to solely cause this discrepancy in flow rates. It appears that other forces are necessary to produce the pressure gradients required to push fluid through the BM and an interesting candidate for such forces is the contraction of vascular smooth muscle cells, as noted in Di Marco et al. (2015) and modeled in detail by (Aldea et al., in preparation).

In conclusion, this study indicates that under physiological conditions arterial pulsations alone are too weak to drive perivascular drainage of ISF through the arterial BM. This result is especially interesting as arterial pulsations have been treated as the most likely candidate for the driving force of perivascular drainage. Other studies, which have considered arterial pulsations as driving mechanisms have either concluded that ISF flow in the reverse direction to the blood flow requires some form of attachment mechanism (Schley et al., 2006; Wang and Olbricht, 2011) or require very specific protein movements giving rise to valve-like behavior (Sharp et al., 2015). Our model suggests a very general valve mechanism that reliably produces net reverse drainage along the BM. While the valve model of the BM provides a physiologically feasible mechanism to ensure net reverse drainage of ISF, it is too small by several orders of magnitude to drive the physiologically observed IPAD. This mathematical model as well as experimental study disproves the widely accepted arterial pulsation hypothesis of perivascular drainage of *A*β from the brain (Schley et al., 2006; Carare et al., 2008; Weller et al., 2009, 2015; Attems et al., 2011; Hawkes et al., 2011; Wang and Olbricht, 2011; Iliff et al., 2012; Morris et al., 2014; Asgari et al., 2015; Sharp et al., 2015) and suggests that other forces must be considered for efficient IPAD and for therapeutic strategies in AD.

## Ethics statement

This study was carried out in accordance with the recommendations of Animals (Scientific Procedures) Act 1986, Section 5, project licence 30/3095.

## Author contributions

AD and GR designed and analyzed BM model and elasticity analysis, AD, NB, and GR designed and analyzed numerical models, RC and MS designed and performed mouse experiments, MG analyzed brain sections and performed image analysis, AD, NB, MS, RC, and GR wrote the paper.

### Conflict of interest statement

The authors declare that the research was conducted in the absence of any commercial or financial relationships that could be construed as a potential conflict of interest. The reviewer AA and handling Editor declared their shared affiliation.

## Supplementary material

All data supporting this study are openly available from Github at https://doi.org/10.5281/zenodo.841142.
