# Supplementary material for: Arterial Pulsations cannot Drive Intramural Periarterial Drainage: Significance for Aβ Drainage
Source: Front Neurosci. 2017 Aug 24;11:475. doi: 10.3389/fnins.2017.00475 (PMC5574214; doi:10.3389/fnins.2017.00475)
Supplement: Supplementary file 1 [file DataSheet1.PDF]

Supplementary Material: Arterial pulsations cannot drive intramural periarterial drainage: Significance for Alzheimer's disease

Alexandra K. Diem, Neil W. Bressloff, Roxana O. Carare, Matthew MacGregor Sharp and Giles Richardson

*Correspondence:
A. K. Diem
A.K.Diem@soton.ac.uk

1 MATHEMATICAL ANALYSIS OF FLOW THROUGH THE BASEMENT MEMBRANE

This section contains the mathematical derivation of the BM valve model equation. The derivation provided here follows are more analytical approach, while the main text contains a more intuitive derivation.

Recall from the main text the dimensionless governing equations and boundary conditions

$$u_z(r,z,t) = -K(p_z)\frac{\partial p(r,z,t)}{\partial z} \tag{1}$$

$$u_r(r,z,t) = -\frac{1}{\varepsilon_L}K(p_z)\frac{\partial p(r,z,t)}{\partial r} \tag{2}$$

$$\frac{\partial u_z(r,z,t)}{\partial z} + \frac{1}{r}\frac{\partial}{\partial r}\left(r u_r(r,z,t)\right) = 0 \tag{3}$$

$$\frac{\partial R_i(z,t)}{\partial t} = u_r(r,z,t) - u_z(r,z,t)\frac{\partial R_i(z,t)}{\partial z} \qquad \text{on } r = R_i(z,t) \tag{4}$$

$$\begin{aligned}\frac{\partial R_i(z,t)}{\partial t} + \varepsilon_R\frac{\partial h(z,t)}{\partial t} &= \\ u_r(r,z,t) - u_z(r,z,t)&\left(\frac{\partial R_i(z,t)}{\partial z} + \varepsilon_R\frac{\partial h(z,t)}{\partial t}\right) \qquad \text{on } r = R_o(z,t).\end{aligned} \tag{5}$$

Note that here the equations are expressed in terms of velocity $\boldsymbol{u}$ instead of flux $\boldsymbol{q}$. Solving Equation 3 for the given boundary condition provides values for the pressure gradient $p_z = \partial p/\partial z$ inside the BM, which is then used to calculate ISF flux through the BM. Performing the variable transformation $(z,r,t) \to (\hat{z},\rho,\hat{t})$, with $0 \le \rho \le h(z,t)$, such that

$$z = \hat{z}$$
$$r = R_i(z,t) + \varepsilon_R \rho$$
$$t = \hat{t}$$

and thus

$$\frac{\partial}{\partial z} = \frac{\partial}{\partial \hat{z}} - \frac{1}{\varepsilon_R}\frac{\partial R_i(z,t)}{\partial \hat{z}}\frac{\partial}{\partial \rho}$$
$$\frac{\partial}{\partial r} = \frac{1}{\varepsilon_R}\frac{\partial}{\partial \rho}$$
$$\frac{\partial}{\partial t} = \frac{\partial}{\partial \hat{t}} - \frac{1}{\varepsilon_R}\frac{\partial R_i(z,t)}{\partial \hat{t}}\frac{\partial}{\partial \rho}.$$

Application of the variable transformation to the governing equations (Equation 1 to Equation 3) results in

$$\hat{u}_{\hat{z}}(\hat{z},\rho,\hat{t}) = -K(p_{\hat{z}} - \frac{1}{\varepsilon_R}\frac{\partial R_i(z,t)}{\partial \hat{z}}p_\rho)\left(\frac{\partial p(\hat{z},\rho,\hat{t})}{\partial \hat{z}} - \frac{1}{\varepsilon_R}\frac{\partial R_i(z,t)}{\partial \hat{z}}\frac{\partial p(\hat{z},\rho,\hat{t})}{\partial \rho}\right) \tag{6}$$

$$\hat{u}_\rho(\hat{z},\rho,\hat{t}) = -\frac{1}{\varepsilon_R\varepsilon_L^2}K(p_{\hat{z}} - \frac{1}{\varepsilon_R}\frac{\partial R_i(z,t)}{\partial \hat{z}}p_\rho)\frac{\partial p(\hat{z},\rho,\hat{t})}{\partial \rho} \tag{7}$$

$$\begin{aligned}\frac{\partial \hat{u}_z(\hat{z},\rho,\hat{t})}{\partial \hat{z}} - \frac{1}{\varepsilon_R}\frac{\partial R_i(z,t)}{\partial \hat{z}}\frac{\partial \hat{u}_z(\hat{z},\rho,\hat{t})}{\partial \rho} &+ \\ \frac{1}{R_i(z,t)+\varepsilon_R\rho}\frac{1}{\varepsilon_R}\frac{\partial}{\partial \rho}\big((R_i(z,t)+\varepsilon_R\rho)\cdot\hat{u}_r(\hat{z},\rho,\hat{t})\big) &= 0,\end{aligned} \tag{8}$$

while the boundary conditions (Equation 4 and Equation 5) become

$$\frac{\partial R_i(z,t)}{\partial \hat{t}} = \hat{u}_\rho(\hat{z},\rho,\hat{t}) - \hat{u}_{\hat{z}}(\hat{z},\rho,\hat{t})\frac{\partial R_i(z,t)}{\partial \hat{z}} \qquad \text{on } \rho = 0 \tag{9}$$

$$\begin{aligned}\frac{\partial R_i(z,t)}{\partial \hat{t}} + \varepsilon_R\frac{\partial h(z,t)}{\partial \hat{t}} &= \\ \hat{u}_\rho(\hat{z},\rho,\hat{t}) - \hat{u}_{\hat{z}}(\hat{z},\rho,\hat{t})\left(\frac{\partial R_i(z,t)}{\partial \hat{z}} + \varepsilon_R\frac{\partial h(z,t)}{\partial \hat{z}}\right) &\qquad \text{on } \rho = h(z,t).\end{aligned} \tag{10}$$

The next step is to look for a solution in powers of the small parameter $\varepsilon_R \ll 1$ by writing

$$p = p_0(\hat{z},\hat{t}) + \varepsilon_R p_1(\hat{z},\rho,\hat{t}) + \mathcal{O}(\varepsilon_R^2)$$

such that the velocity components become

$$\hat{u}_{\hat{z}}(\hat{z},\rho,\hat{t}) = -K(p_{0\hat{z}} - \frac{\partial R_i(z,t)}{\partial \hat{z}}p_{1\rho})\left(\frac{\partial p_0(\hat{z},\hat{t})}{\partial \hat{z}} - \frac{\partial R_i(z,t)}{\partial \hat{z}}\frac{\partial p_1(\hat{z},\rho,\hat{t})}{\partial \rho}\right) \tag{11}$$

$$\hat{u}_\rho(\hat{z},\rho,\hat{t}) = -\frac{1}{\varepsilon_L^2}K(p_{0\hat{z}} - \frac{1}{\varepsilon_R}\frac{\partial R_i(z,t)}{\partial \hat{z}}p_{1\rho})\frac{\partial p_1(\hat{z},\rho,\hat{t})}{\partial \rho}. \tag{12}$$

Applying this substitution to the governing equations and boundary conditions and letting $\varepsilon_R \to 0$ leads to a simplified model in terms of the leading order pressure term $p_0(\hat{z},\hat{t})$. The governing equations are now Equations 11, 12 and the continuity equation

$$\frac{\partial \hat{u}_{0\hat{z}}(\hat{z},\hat{t})}{\partial \hat{z}} - \frac{\partial R_i(z,t)}{\partial \hat{z}}\frac{\partial \hat{u}_{1\hat{z}}(\hat{z},\rho,\hat{t})}{\partial \rho} + \frac{\partial \hat{u}_{1\rho}(\hat{z},\rho,\hat{t})}{\partial \rho} + \frac{\hat{u}_{0\rho}(\hat{z},\hat{t})}{R_i(z,t)} = 0 \tag{13}$$

and the boundary conditions become

$$\frac{\partial R_i(z,t)}{\partial \hat{t}} = \hat{u}_{0\rho}(\hat{z},\hat{t}) - \hat{u}_{0\hat{z}}(\hat{z},\hat{t})\frac{\partial R_i(z,t)}{\partial \hat{z}} \qquad \text{on } \rho = 0 \tag{14}$$

$$\frac{\partial h(z,t)}{\partial \hat{t}} = \hat{u}_{1\rho}(\hat{z},\rho,\hat{t}) - \hat{u}_{1\hat{z}}(\hat{z},\rho,\hat{t})\frac{\partial h(z,t)}{\partial \hat{z}} \qquad \text{on } \rho = h(z,t). \tag{15}$$

To obtain a solution Equation 13 is integrated over the width of the BM w. r. t.ρ

$$\int_0^{h(z,t)} \left(\frac{\partial \hat{u}_{0\hat{z}}(\hat{z},\hat{t})}{\partial \hat{z}} - \frac{\partial R_i(z,t)}{\partial \hat{z}}\frac{\partial \hat{u}_{1\hat{z}}(\hat{z},\rho,\hat{t})}{\partial \rho} + \frac{\partial \hat{u}_{1\rho}(\hat{z},\rho,\hat{t})}{\partial \rho} + \frac{\hat{u}_{0\rho}(\hat{z},\hat{t})}{R_i(z,t)}\right) d\rho = 0$$

and yields

$$h(z,t)\left(\frac{\partial \hat{u}_{0\hat{z}}(\hat{z},\hat{t})}{\partial \hat{z}} + \frac{\hat{u}_{0\rho}(\hat{z},\hat{t})}{R_i(z,t)}\right) + \left[\hat{u}_{1\rho}(\hat{z},\rho,\hat{t}) - \frac{\partial R_i(z,t)}{\partial \hat{z}}\hat{u}_{1\hat{z}}(\hat{z},\rho,\hat{t})\right]_0^{h(z,t)} = 0. \tag{16}$$

The remaining terms in Equation 16 can be evaluated using the boundary conditions Equations 14 and 15. Dropping subscripts the model equation is

$$\frac{\partial}{\partial \hat{t}}\left(R_i(z,t)\cdot h(z,t)\right) + \frac{\partial}{\partial \hat{z}}\left(R_i(z,t)\cdot h(z,t)\cdot u(\hat{z},\hat{t})\right) = 0 \tag{17}$$

with

$$u = -K(p_{\hat{z}})\frac{\partial p(\hat{z},\hat{t})}{\partial \hat{z}}$$

2 STRESS AND STRAIN IN THE ARTERY WALL

All calculations are carried out neglecting body forces such as gravity. Under this assumption stress can only be induced in a solid by applying a force to one of its boundaries. Stress is measured in units of force per area ($\mathrm{N\,m^{-2}}$, more commonly denoted as Pa). Strain, on the other hand, is a dimensionless quantity that describes the amount of deformation of a solid. Usually, one is interested in strain due to stress, i. e.the amount of deformation of a solid due to some force applied to one or more of its boundaries. This relationship between stress and strain is captured in the stress-strain curve, which is unique for every material.

Here, strain is evaluated as the result of stress on the inner boundary of the artery wall. Stress is induced by the pressure pulse running through the artery and is directly proportional to pressure. First, a derivation for obtaining radial stress at any point inside the artery wall is provided. Following this derivation, a fluid-filled annulus representing the BM is introduced inside the artery wall. It is shown that for a sufficiently small BM the fluid-filled annulus can be shrunk to an infinitesimally thin ring. Due to this observation the pressure of ISF inside the BM can be directly obtained from the stress in the artery wall. This pressure serves as an input to the model of the BM derived in Supplementary Section 1.

2.1 Plane Strain in an Annulus

Consider an annulus (Supplementary Figure 1) in the (r,θ) plane of a cylindrical coordinate system on whose inner boundary ($r = a$) some pressure P is applied. This corresponds to a cross-section of an idealised artery whose centre is aligned with the z-axis of the coordinate system. For simplicity P

is assumed to be continuous and uniform everywhere on the inner boundary of the annulus. Cauchy's momentum equations (1), with displacement $\boldsymbol{v}$ independent of z and t, are

$$0 = \frac{1}{r}\frac{\partial}{\partial r}(r\tau_{rr}) + \frac{1}{r}\frac{\partial \tau_{r\theta}}{\partial \theta} - \frac{\tau_{\theta\theta}}{r} \tag{18}$$

$$0 = \frac{1}{r}\frac{\partial}{\partial r}(r\tau_{r\theta}) + \frac{1}{r}\frac{\partial \tau_{\theta\theta}}{\partial \theta} - \frac{\tau_{\theta\theta}}{r}. \tag{19}$$

The components of the stress tensor τ are

$$\tau_{rr} = (\lambda_L + 2\mu_L)e_{rr} + \lambda_L e_{\theta\theta} \tag{20}$$

$$\tau_{\theta\theta} = \lambda_L e_{rr} + (\lambda_L + 2\mu_L)e_{rr} \tag{21}$$

$$\tau_{r\theta} = 2\mu_L e_{r\theta} \tag{22}$$

with material specific Lamé coefficients λ_L and μ_L and strain tensor e whose components are

$$e_{rr} = \frac{\partial v_r}{\partial r} \tag{23}$$

$$e_{\theta\theta} = \frac{1}{r}\left(\frac{\partial v_\theta}{\partial \theta} + v_r\right) \tag{24}$$

$$e_{r\theta} = \frac{1}{r}\frac{\partial v_r}{\partial \theta} + \frac{\partial v_\theta}{\partial r} - \frac{v_\theta}{r}. \tag{25}$$

As mentioned before pressure is directly proportional to stress and therefore the boundary conditions

$$\tau_{rr} = -P \qquad \text{on } r = a \tag{26}$$

$$\tau_{rr} = 0 \qquad \text{on } r = b \tag{27}$$

apply. Equation 27 is often also referred to as a zero traction boundary condition. Two more boundary conditions are obtained from the fact that the annulus is considered to be radially symmetric and therefore

$$\tau_{r\theta} = 0 \qquad \text{on } r = a \tag{28}$$

$$\tau_{r\theta} = 0 \qquad \text{on } r = b. \tag{29}$$

Equation 18 and Equation 19 and the boundary conditions Equation 26, Equation 28 and Equation 27 are nondimensionalised using the dimensionless parameters in Supplementary Table 1

$$0 = \frac{\tau_{rr}}{r} + \frac{\partial \tau_{rr}}{\partial r} + \frac{\varepsilon_L}{r}\frac{\partial \tau_{r\theta}}{\partial \theta} - \frac{\tau_{\theta\theta}}{r} \tag{30}$$

$$0 = \frac{\tau_{r\theta}}{r} + \frac{\partial \tau_{r\theta}}{\partial r} + \frac{1}{\varepsilon_L r}\frac{\partial \tau_{\theta\theta}}{\partial \theta} - \frac{\tau_{\theta\theta}}{r}. \tag{31}$$

$$\tau_{rr} = (1 + 2\varepsilon_L)\frac{\partial v_r}{\partial r} + \frac{1}{r}\frac{\partial v_\theta}{\partial \theta} + \frac{v_r}{r} \tag{32}$$

$$\tau_{\theta\theta} = \frac{\partial v_r}{\partial r} + (1 + 2\varepsilon_L)\left(\frac{1}{r}\frac{\partial v_\theta}{\partial \theta} + \frac{v_r}{r}\right) \tag{33}$$

$$\tau_{r\theta} = \frac{1}{r}\frac{\partial v_r}{\partial \theta} + \frac{\partial v_\theta}{\partial r} - \frac{v_\theta}{r} \tag{34}$$

$$\tau_{rr} = -P \qquad \text{on } r = a \tag{35}$$

$$\tau_{r\theta} = 0 \qquad \text{on } r = a \tag{36}$$

$$\tau_{rr} = \tau_{r\theta} = 0 \qquad \text{on } r = b. \tag{37}$$

Expanding the displacement and stress tensor components in terms of the small parameter ε_L leads to

$$v_r = v_{r,0} + \varepsilon_L v_{r,1} + \mathcal{O}(\varepsilon_L^2)$$
$$v_\theta = v_{\theta,0} + \varepsilon_L v_{\theta,1} + \mathcal{O}(\varepsilon_L^2)$$
$$\tau_{rr} = \tau_{rr,0} + \varepsilon_L \tau_{rr,1} + \mathcal{O}(\varepsilon_L^2)$$
$$\tau_{\theta\theta} = \tau_{\theta\theta,0} + \varepsilon_L \tau_{\theta\theta,1} + \mathcal{O}(\varepsilon_L^2)$$
$$\tau_{r\theta} = \tau_{r\theta,0} + \varepsilon_L \tau_{r\theta,1} + \mathcal{O}(\varepsilon_L^2).$$

It is now shown that the leading order terms $\tau_{rr,0}$ and $\tau_{r\theta,0}$ vanish. This follows from the near incompressibility of the arterial wall

$$\nabla \cdot \boldsymbol{v} = \frac{\partial v_{r,0}}{\partial r} + \frac{v_{r,0}}{r} + \frac{1}{r}\frac{\partial v_{\theta,0}}{\partial \theta} = 0. \tag{38}$$

The leading order terms in Equation 32 to Equation 34 are

$$\tau_{rr,0} = \frac{\partial v_{r,0}}{\partial r} + \frac{1}{r}\frac{\partial v_{\theta,0}}{\partial \theta} + \frac{v_{r,0}}{r} \tag{39}$$

$$\tau_{\theta\theta,0} = \frac{\partial v_{r,0}}{\partial r} + \frac{1}{r}\frac{\partial v_{\theta,0}}{\partial \theta} + \frac{v_{r,0}}{r} \tag{40}$$

$$\tau_{r\theta,0} = \frac{1}{r}\frac{\partial v_{r,0}}{\partial \theta} + \frac{\partial v_{\theta,0}}{\partial r} - \frac{v_{\theta,0}}{r}. \tag{41}$$

Both $\tau_{rr,0}$ and $\tau_{r\theta,0}$ are equal to Equation 38 and therefore indeed vanish. The governing equations Equation 30 and Equation 31 become

$$0 = \frac{\tau_{rr,1}}{r} + \frac{\partial \tau_{rr,1}}{\partial r} + \frac{\varepsilon_L}{r}\frac{\partial \tau_{r\theta,0}}{\partial \theta} + \frac{\varepsilon_L^2}{r}\frac{\partial \tau_{r\theta,1}}{\partial \theta} - \frac{\tau_{\theta\theta,1}}{r} \tag{42}$$

$$0 = \frac{\tau_{r\theta,0}}{r} + \varepsilon_L \frac{\tau_{r\theta,1}}{r} + \frac{\partial \tau_{r\theta,0}}{\partial r} + \varepsilon_L \frac{\partial \tau_{r\theta,1}}{\partial r} + \frac{1}{\varepsilon_L r}\frac{\partial \tau_{\theta\theta,1}}{\partial \theta} - \frac{\tau_{r\theta,0}}{r} - \varepsilon_L \frac{\tau_{r\theta,1}}{r} \tag{43}$$

and the boundary conditions are

$$\tau_{rr,1} = -P \qquad \text{on } r = a \tag{44}$$

$$\tau_{r\theta,1} = 0 \qquad \text{on } r = a \tag{45}$$

$$\tau_{rr,1} = \tau_{r\theta,1} = 0 \qquad \text{on } r = b. \tag{46}$$

Letting $\varepsilon_L \to 0$ and dropping subscripts Equation 43 vanishes completely and Equation 42 reduces to

$$0 = \frac{\tau_{rr}}{r} + \frac{d\tau_{rr}}{dr} - \frac{\tau_{\theta\theta}}{r} \tag{47}$$

with boundary conditions

$$\tau_{rr} = -P \qquad \text{on } r = a \tag{48}$$

$$\tau_{rr} = 0 \qquad \text{on } r = b. \tag{49}$$

The system of Equation 47 to Equation 49 now only depends on r. This makes sense as pressure is applied uniformly across the boundary on $r = a$ and therefore does not vary in the θ-direction. To solve for τ_{rr} an Airy stress function $\mathcal{A}(r)$ is used that satisfies the biharmonic equation

$$\nabla^4 \mathcal{A}(r) = 0. \tag{50}$$

Similar to a stream function in fluid dynamics the Airy stress function is a potential function for the stresses in a solid and is only defined to within a linear function in r (1). Again, for readability the notation $\mathcal{A} = \mathcal{A}(r)$ is used from now on. To find a solution for $\mathcal{A}$ one can begin by defining

$$\tau_{rr} = \frac{1}{r}\frac{d\mathcal{A}}{dr} \tag{51}$$

and from Equation 47

$$\tau_{\theta\theta} = \frac{d^2\mathcal{A}}{dr^2} \tag{52}$$

follows. Expanding Equation 50 the biharmonic equation for $\mathcal{A}$ is

$$\frac{1}{r}\frac{d}{dr}\left(r\frac{d}{dr}\left(\frac{1}{r}\frac{d}{dr}\left(r\frac{d\mathcal{A}}{dr}\right)\right)\right) = 0, \tag{53}$$

which has a solution of the form

$$\mathcal{A} = k_1 r^2 + k_2 + k_3 r^2 \log(r) + k_4 \log(r). \tag{54}$$

To solve for the unknown constants $k_1, \ldots, k_4$ the boundary conditions Equation 48 and Equation 49 are used. At $r = a$ the stress on the boundary is determined by pressure P such that

$$\frac{1}{r}\frac{d\mathcal{A}}{dr} = -P \qquad \text{on } r = a. \tag{55}$$

Equation 49 means that no surface traction is applied on the boundary $r = b$. Surface traction $t(s)$ of arc length s is given by

$$t(s) = \frac{d}{ds}\left(\frac{d\mathcal{A}}{dr}\right). \tag{56}$$

Integrating w. r. t.s and applying $t(s) = 0$ gives

$$\frac{d\mathcal{A}}{dr} = C \qquad \text{on } r = b, \tag{57}$$

where C is the constant of integration. Remembering that $\mathcal{A}$ is only defined to within a linear function in r one can, without loss of generality, define $C = 0$. Thus, Equation 57 becomes

$$\frac{d\mathcal{A}}{dr} = 0 \qquad \text{on } r = b. \tag{58}$$

To obtain a condition for $\mathcal{A}$ on the boundary $r = b$ rewrite

$$\frac{d\mathcal{A}}{dr} = \frac{d\mathcal{A}}{ds}\boldsymbol{e}_t + \frac{d\mathcal{A}}{dn}\boldsymbol{e}_n, \tag{59}$$

where $\boldsymbol{e}_t$ and $\boldsymbol{e}_n$ are the unit tangent and outer normal vectors on the boundary. Integrating w. r. t.s and using Equation 58 yields

$$\mathcal{A} = 0 \qquad \text{on } r = b. \tag{60}$$

Thus far there are three boundary conditions (Equation 55, Equation 58 and Equation 60) for the four unknown constants in Equation 54. The fourth condition required to solve Equation 54 is called a compatibility condition and is necessary to ensure that the displacement is unique for a certain strain. This condition is not automatically satisfied since there are two stress components τ_{rr} and $\tau_{\theta\theta}$ for just one displacement component $v_r(r)$. Differentiating τ_{rr} and $\tau_{\theta\theta}$ and adding them yields the compatibility condition

$$\frac{d^3\mathcal{A}}{dr^3} + \frac{1}{r}\frac{d^2\mathcal{A}}{dr^2} - \frac{1}{r^2}\frac{d\mathcal{A}}{dr} = 0. \tag{61}$$

which is satisfied only if $k_3 = 0$. Using the boundary conditions to determine the remaining constants results in the solution

$$\mathcal{A}(r) = \frac{Pa^2b^2}{a^2 - b^2} \cdot \log\left(\frac{r}{b}\right) + \frac{Pa^2}{2(a^2 - b^2)}\left(b^2 - r^2\right) \tag{62}$$

for a given radial position r. The stress components evaluate to

$$\tau_{rr} = \frac{Pa^2}{b^2 - a^2}\left(1 - \frac{b^2}{r^2}\right) \tag{63}$$

$$\tau_{\theta\theta} = \frac{Pa^2}{b^2 - a^2}\left(1 + \frac{b^2}{r^2}\right). \tag{64}$$

The Airy stress function approach provides a solution for a material with a linear stress-strain relation.

2.2 Validation of the Plane Strain Model

The plane strain model for calculating the stresses inside an artery makes a number of simplifying assumptions:

1. Stress is calculated without regard for the longitudinal z-direction.
2. The artery wall is a linear elastic material.
3. Pressure inside the artery is uniform across the lumen area.

The validity of these assumptions shall be addressed in this section before proceeding with an adaptation of the plane strain model to obtain ISF pressure inside the BM.

The first assumption arises from the fact that blood vessels are longitudinally tethered, i. e.both ends of the arterial tree are attached to the heart, which limits the amount of stretch they can undergo along the z-direction. This assumption has been used in the literature and the same equations have successfully been used to accurately describe blood flow dynamics by others (2; 3; 5; 4). To evaluate the second assumption one has to recognise that the artery wall actually is a viscoelastic material. This means that there is a time delay between an increase in blood pressure (i. e.increased radial stress on the artery wall) and the corresponding increase in vessel diameter. This effect cannot accurately be captured by a purely elastic material. However, (6) showed that this time lag is mainly an experimental artefact and have developed a model to correct experimental measurements for this artefact. No other publication could be found to investigate this hypothesis, but results from other blood flow simulation studies show that this simple model appears to suffice to accurately model blood flow dynamics.

To confirm the second and address the third assumption a simple model was set up in the finite element multiphysics modelling software COMSOL 5.1. The geometry is a 2D axisymmetric tube of length L_0 with radius R_0 surrounded by a wall of thickness h_0. Flow inside the tube is governed by the Navier-Stokes equations for laminar flow

$$\rho(\boldsymbol{u} \cdot \nabla)\boldsymbol{u} = \nabla \cdot \left(-p\boldsymbol{I} + \mu \left(\nabla \boldsymbol{u} + (\nabla \boldsymbol{u})^T\right)\right) + \boldsymbol{F}_s \tag{65}$$

$$\rho \nabla \cdot \boldsymbol{u} = 0 \tag{66}$$

with $\boldsymbol{I}$ identity matrix and $\boldsymbol{F}_s$ some external body force. The dynamics of the wall are governed by either the equations for linear elasticity or hyperelasticity. In both cases the stress relates to $\boldsymbol{F}_s$ via

$$-\nabla \sigma = \boldsymbol{F}_s \nu_s \tag{67}$$

with σ stress tensor and ν_s Poisson's ratio. In the case of the linear elastic model stress is governed by Hooke's law

$$\sigma = \sum_i \sum_j C_{ij} \varepsilon_{\text{el},ji} \tag{68}$$

$$\varepsilon_{\text{el}} = \frac{1}{2}\left((\nabla \boldsymbol{v})^T + \nabla \boldsymbol{v} + (\nabla \boldsymbol{v})^T \nabla \boldsymbol{v}\right) \tag{69}$$

with C elasticity tensor and ε_{el} elastic strain tensor. In the case of nonlinear elasticity stress is governed by a Neo-Hookean material model, which has been shown to provide a good fit for the mechanical response of arterial elastin (7; 8), where stress is given as

$$\sigma = J^{-1}\boldsymbol{F}_s\boldsymbol{S}_s\boldsymbol{F}_s^T \tag{70}$$

with $J = \det(\boldsymbol{F}_s)$ and $\boldsymbol{S}_s = \partial W_s/\partial\varepsilon_{el}$ second Piola-Kirchoff stress. For a nearly incompressible neo-Hookean material the strain energy density function is

$$W_s = W_{\text{iso}} + W_{\text{vol}} = \frac{1}{2}\mu_s(\bar{I}_1 - 3) + \frac{1}{2}K_s(J_{\text{el}} - 1)^2, \tag{71}$$

where μ_s initial shear modulus, $\bar{I}_1$ first principal invariant of the symmetric right Cauchy-Green tensor, K_s initial bulk modulus and J_{el}. The boundary between the lumen and artery wall implements a two-way coupling between the two domains: In the fluid domain pressure is determined by the normal stress on the wall

$$\sigma \cdot \boldsymbol{n} = \left(-p\boldsymbol{I} + \mu\left(\nabla\boldsymbol{u} + (\nabla\boldsymbol{u})^T\right)\right) \cdot \boldsymbol{n}, \tag{72}$$

while the rate of displacement of the artery wall is determined by the fluid velocity

$$\frac{\partial\boldsymbol{v}}{\partial t} = \boldsymbol{u}. \tag{73}$$

Because the artery is tethered displacement of the artery wall is restricted to the z-direction at open ends

$$\boldsymbol{v} \cdot \boldsymbol{n} = 0. \tag{74}$$

At the fluid inlet a velocity is applied, while at the outlet pressure is applied. The parameter values used for the simulations are listed in Supplementary Table 2.

The results of this simulation are shown in Supplementary Figure 2. There is good agreement between the hyperelastic and linear elastic material while the Airy stress function overestimates hoop stress ($\tau_{\theta\theta}$) at the higher end of the pressure range. This is acceptable as an overestimation of stress would lead to an overestimation of ISF pressure inside the BM. If ISF pressure inside the BM is too small to drive significant flow when stress is estimated using the Airy stress function then it will also be too small when using a hyperelastic numerical simulation. Furthermore, only radial stress is required for the calculation of ISF pressure and the overall agreement between the Airy stress function and the simulation results is found to be suitably good for radial stress. Therefore the Airy stress function calculation was used for the remainder of this thesis.

2.3 Plane Strain in an Artery Cross-Section with BM

The Airy stress function derived in Section 2.1 can also be used to determine the pressure of ISF in the BM. For simplicity the BM shall be treated as a thin fluid-filled empty space. This approximation is considered valid for the fluid part of a porous medium if the fluid-filled space is small enough. The BM is only approximately $150\,\text{nm}$ in thickness, which is roughly three orders of magnitude smaller than the thickness of the artery wall of the MCA and therefore, the approximation is considered valid. Two independent Airy stress functions $\mathcal{A}_1(r)$ and $\mathcal{A}_2(r)$ are thus used to describe the artery wall parts

surrounding the BM (Supplementary Figure 3). Following the analysis in Section 2.1 these are

$$\mathcal{A}_1 = k_1 r^2 + k_2 + k_3 \log(r) \tag{75}$$

$$\mathcal{A}_2 = k_4 r^2 + k_5 + k_6 \log(r) \tag{76}$$

and as before the boundary conditions are

$$\frac{1}{r}\frac{d\mathcal{A}_1}{dr} = -P \qquad \text{on } r = a_1 \tag{77}$$

$$\mathcal{A}_2 = \frac{d\mathcal{A}_2}{dr} = 0 \qquad \text{on } r = b_2. \tag{78}$$

The pressure of ISF P_{BM} is required to be continuous and uniform and therefore

$$\frac{d\mathcal{A}_1}{dr} = \frac{d\mathcal{A}_2}{dr} \qquad \text{on } r = b_1, a_2 \tag{79}$$

applies.

The claim that, given a sufficiently thin BM, a single Airy stress function can be used to model the artery wall like in Section 2.1 and the ISF pressure can then be directly obtained from the stress in the wall at the location of the BM shall be proven here. Because ISF is an incompressible fluid its area A_f has to remain constant

$$A_f = \pi\left((a_2 + v_r(a_2))^2 - (b_1 + v_r(b_1))^2\right) = const. \tag{80}$$

Eliminating π and the small square terms in v_r yields

$$2a_2 v_r(a_2) - 2b_1 v_r(b_1) = b_1^2 - a_2^2. \tag{81}$$

As the fluid part of the BM is considered to be very thin one can take the limit

$$\lim_{b_1 \to a_2} 2a_2 v_r^+(a_2) - 2b_1 v_r^-(b_1) = b_1^2 - a_2^2. \tag{82}$$

and confirm

$$v_r^+(a_2) = v_r^-(b_1). \tag{83}$$

Therefore $\mathcal{A}_1$ and $\mathcal{A}_2$ must be continuous across the BM such that

$$\mathcal{A}_1 = \mathcal{A}_2 \qquad \text{on } r = b_1, a_2. \tag{84}$$

Therefore the claim is proven and a single Airy stress function $\mathcal{A} = \mathcal{A}_1 + \mathcal{A}_2$ can be used to describe the artery wall. The ISF pressure can then be found by evaluating τ_{rr} (Equation 63) at $r = b_1$

$$P_{\text{BM}} = -\frac{Pa_1^2}{b_2^2 - a_1^2}\left(1 - \frac{b_2^2}{b_1^2}\right). \tag{85}$$

REFERENCES

[1]Howell P, Kozyreff G and Ockendon J (2008) *Applied Solid Mechanics.* Cambridge University Press, Cambridge, UK.

[2]Olufsen MS et al. (2000) Numerical Simulation and Experimental Validation of Blood Flow in Arteries with Structured-Tree Outflow Conditions. *Ann. Biomed. Eng.* 28(11):1281–1299.

[3]Kolachalama V, Bressloff NW, Nair PB, Shearman CP (2007) Predictive Haemodynamics in a One-Dimensional Carotid Artery Bifurcation. Part I: Application to Stent Design. *IEEE Trans. Biomed. Eng.* 54(5):802–812.

[4]Cousins W, Gremaud PA (2014) Impedance boundary conditions for general transient hemodynamics. *Int. J. Numer. Method Biomed. Eng.*

[5]Devault K and Gremaud A (2008) Blood Flow in the Circle of Willis: Modeling and Calibration. *Multiscale Model. Simul.* 7(2):888–909.

[6]Tardy Y, Meister JJ, Perret F, Brunner HR and Arditi M (1991) Non-invasive estimate of the mechanical properties of peripheral arteries from ultrasonic and photoplethysmographic measurements. *Clin. Phys. Physiol. Meas.* 12(1):39–54.

[7]Holzapfel GA and Weizsacker HW (1998) Biomechanical behaviour of the arterial wall and its numerical characterization. *Comput. Biol. Med.* 28(4):377–392.

[8]Gundiah N, Ratcliffe MB and Pruitt LA (2007) Determination of strain energy function for arterial elastin: Experiments using histology and mechanical tests. *J. Biomech.* 40(3):586–594.

Supplementary Table 1. Dimensionless variables and parameters used to nondimensionalise the equations of the stress tensor components of an inflated annulus (Equation 20, Equation 21 and Equation 22).

Parameter	Physical meaning
L	length
R	radius
V	tangential displacement
λ_L	first Lamé coefficient
μ_L	second Lamé coefficient
$\varepsilon_L = R/L \ll 1$	radius-to-length ratio
Variable	
$r \sim R = \varepsilon_L L$	radius
$v_r \sim \varepsilon_L V$	radial displacement
$v_\theta \sim V$	tangential displacement
$\tau_{rr} \sim \lambda_L V/L$	radial stress
$\tau_{\theta\theta} \sim \lambda_L V/L$	hoop stress
$\tau_{r\theta} \sim \mu_L V/L$	
$P \sim \lambda_L V/L$	blood pressure

Supplementary Table 2. Parameters used in the linear and hyperelastic FE simulation of an artery.

Parameter	Value	Physical meaning
R	$0.134\,\mathrm{cm}$	vessel radius
L	$1.2\,\mathrm{cm}$	vessel length
h	$R/4$	wall thickness
u_{in}	$0.6\,\mathrm{cm\,s^{-1}}$	inlet velocity
p_{out}	80 / 100 / 120 mmHg	outlet pressure
μ	$4.88 \times 10^{-3}\,\mathrm{Pa\,s}$	dynamic viscosity of blood
ρ	$1.06\,\mathrm{g\,m^{-3}}$	blood density
ρ_s	$0.9\,\mathrm{g\,m^{-3}}$	artery wall density
Linear elastic material properties		
E	$7.37 \times 10^5\,\mathrm{Pa}$	Young's modulus
ν_s	0.495	Poisson's ratio
Hyperelastic material properties		
μ_s	$E/(2(1+\nu))$	initial shear modulus
K_s	$E/(3(1-2\nu))$	initial bulk modulus

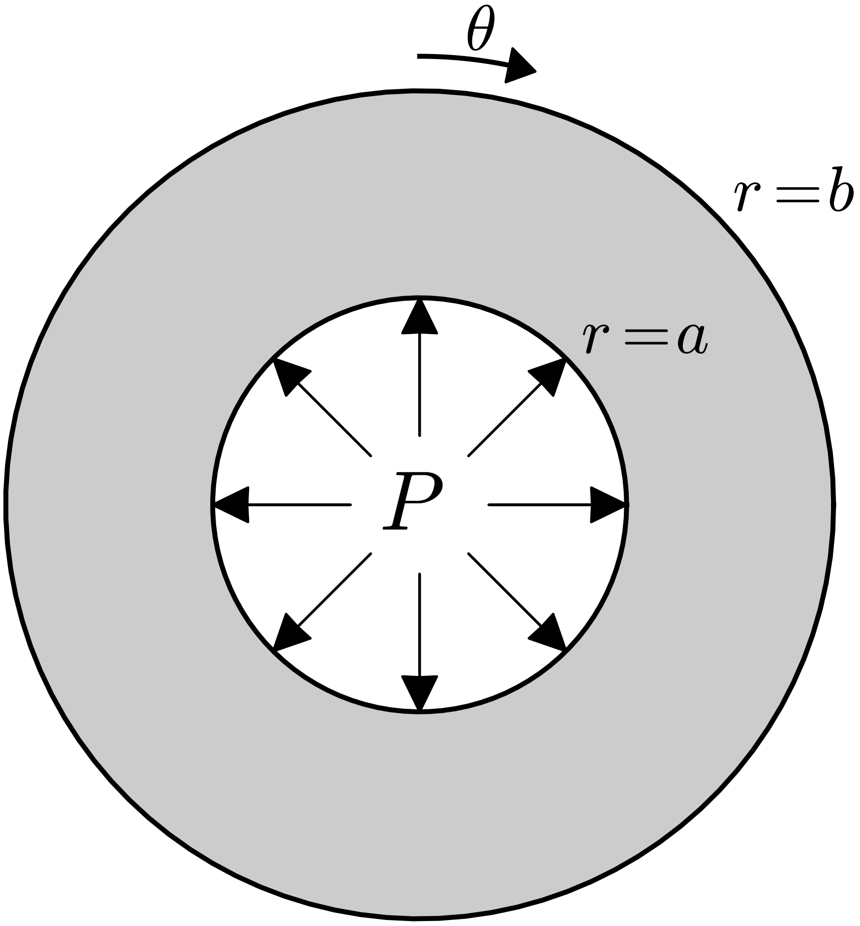

Supplementary Figure 1. Schematic representation of a linear elastic annulus in the (r, θ) plane inflated by an internal pressure P.

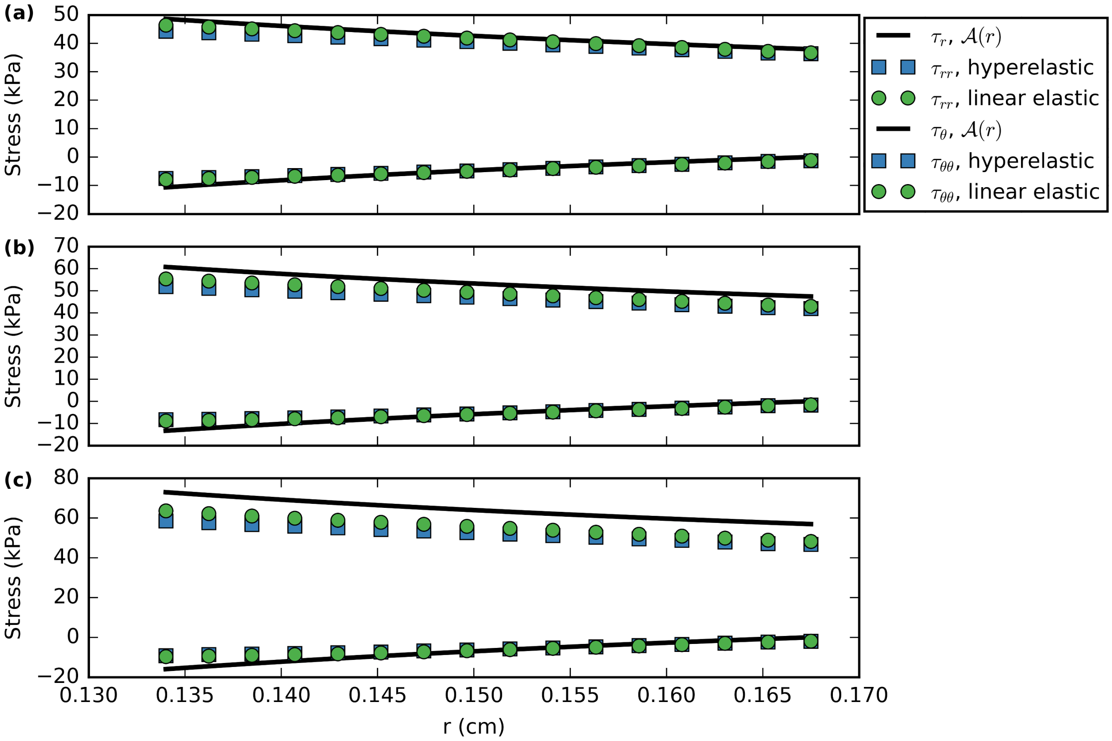

Supplementary Figure 2. Comparison of radial and hoop stresses in an artery wall using the Airy stress function (black line) or an FE simulation with linear (green circles) or hyperelastic (blue squares) material at various blood pressures. (a) $p = 80\,\mathrm{mmHg}$, (b) $p = 100\,\mathrm{mmHg}$, (c) $p = 120\,\mathrm{mmHg}$.

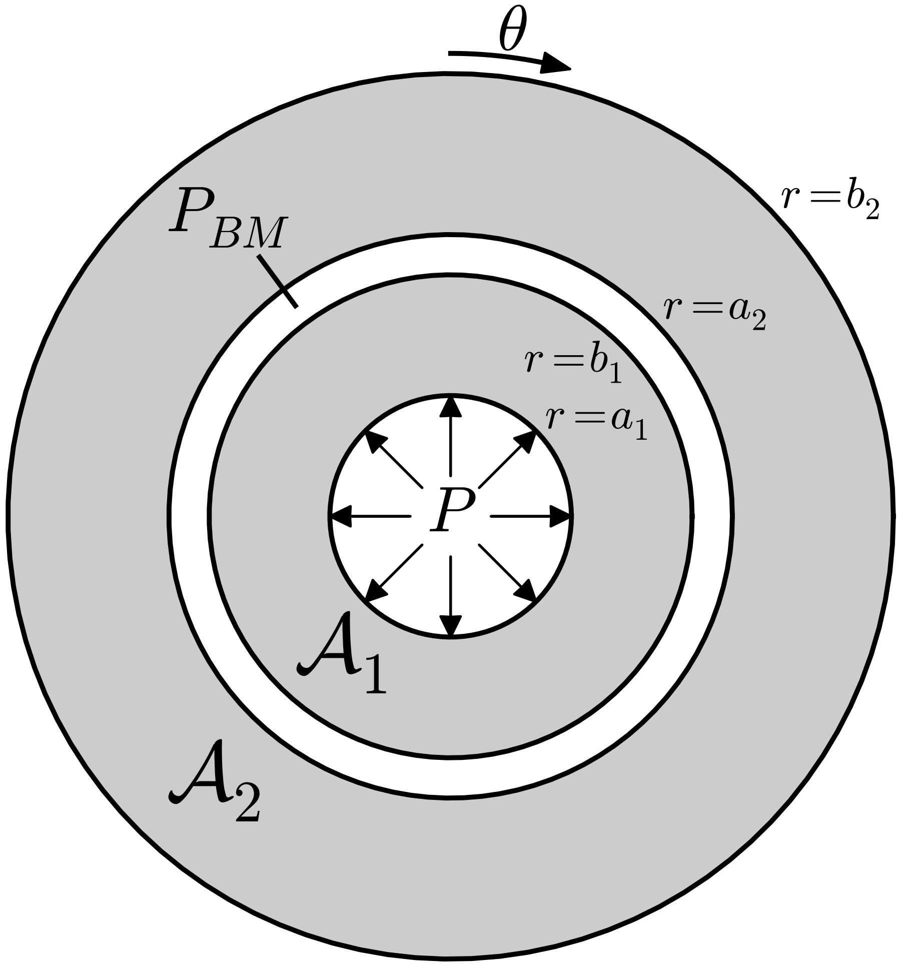

Supplementary Figure 3. Schematic representation of the elastic model of an artery cross-section with BM inflated by an internal pressure P. For simplicity the BM is approximated by a fluid-filled space at some radial position r. The artery wall is then split into two separate parts, which are described by two independent Airy stress functions $\mathcal{A}_1$ and $\mathcal{A}_2$.
